# Informing reform: The views of legal professionals on the unique aspects of Scottish Law

**DOI:** 10.1177/0025802421992913

**Published:** 2021-02-17

**Authors:** Lee John Curley, James Munro, Lara A Frumkin, Jim Turner

**Affiliations:** Faculty of Arts and Social Sciences, School of Psychology and Counselling, The Open University, UK

**Keywords:** ‘Not proven’ verdict, 15-person jury, qualified majority verdict system, simple majority verdict system, Scottish legal system, advocate attitudes

## Abstract

The unique Scottish legal system stands apart from the better-known Anglo-American legal system, with variations relating to jury size (15 vs. 12), the number of verdicts available (3 vs. 2) and majority size (simple majority vs. unanimous). At present, only a handful of investigations have explored the effects of the Scottish ‘not proven’ verdict on jurors, and only a single study has explored the combined impact of the unique elements of the Scottish legal system on juror and jury decision making. The current study is the first to investigate the views of Scottish legal professionals on the three-verdict system, 15-person jury and simple majority verdict system. The aim of the study is to inform public and political debate, involve legal stakeholders in policy changes and decision making and compare legal professionals’ views with findings from previously conducted juror studies. Seventy-eight legal professionals took part in an online survey which asked for ratings and open responses on their attitudes to the Scottish (a) three-verdict system, (b) 15-person jury and (c) simple majority system. The results highlighted strong positive attitudes towards the ‘not proven’ verdict (particularly in a binary-verdict system of proven and not proven), 15-person juries and both the simple and qualified majority verdict systems. There was minimal support for reform towards an Anglo-American system. Instead, the reforms preferred by the legal professionals would be to require a qualified majority of 12/15 jurors, and to use a binary-verdict system of proven and not proven.

## Introduction

Scotland has a unique legal system in regard to juries. Unlike the rest of the UK and countries such as the USA which have two verdicts available to jurors (i.e. ‘guilty’ and ‘not guilty’), Scotland has three verdicts.^1^ Juries in Scotland can give verdicts of ‘guilty’, ‘not guilty’ or ‘not proven’. The ‘not proven’ verdict functions as an acquittal, although it has never been legally defined.^1^ Despite this, jurors are told that individuals who are given a ‘not proven’ verdict are acquitted in the same manner as a ‘not guilty’ verdict. Juries in Scotland are made up of 15 jurors rather than the 12 found in other legal systems based on the English model.^2^ In addition, juries in Scotland do not need to reach a unanimous verdict or even a qualified majority (10 jurors in agreement). Rather, a decision can be given by a simple majority of jurors (8/15) agreeing on the same verdict.^2^

The aim of the current study – the first of its kind in Scotland – was to investigate legal professionals’ attitudes to each of these specific jury factors. Legal professionals in Scotland encounter the unique elements of the Scottish legal regularly, often over a period of years or decades, and witness hundreds of jurors engaged with the unique aspects mentioned above. There is no other group of individuals who have more exposure to the elements discussed in the current paper. Therefore, we investigated Scottish legal professionals’ views in order to: (a) inform public and political debate, (b) involve legal stakeholders in policy changes and decision making and (c) compare legal professionals’ views to findings from previously conducted juror studies.

In recent years, each of the factors mentioned have faced public scrutiny. For example, there have been suggestions that the ‘not proven’ verdict may decrease convictions and increase the chances of *truly* guilty individuals being acquitted.^3^ A campaign for the removal of the ‘not proven’ verdict has been led by Miss M, an activist against the ‘not proven’ verdict, as the said verdict is used more frequently in rape trials than other criminal trials.^3^ Indeed, the ‘not proven’ verdict has been scrutinised for almost 200 years, with Sir Walter Scott naming it ‘the bastard verdict’.^4^

Despite Scottish jurisprudence having such differences from other (more extensively researched) legal systems for centuries, and the efficacy of the ‘not proven’ verdict being debated for almost as long, only recently have academics begun investigating how the unique factors of Scottish law may affect juror and jury decision making. The first experimental paper considering them was published in 2007,^5^ 180 years after Sir Walter Scott’s critical epithet was bestowed.

Four published studies have empirically investigated the effect that the availability of the ‘not proven’ verdict has on juror/jury decision making. Smithson et al.^5^ used a case vignette method in which participants acting as jurors were required to give decisions using a two-verdict (guilty vs. not guilty) and three-verdict (including not proven) system. Their main finding was that the availability of the ‘not proven’ verdict reduced the proportion of ‘not guilty’ verdicts, without affecting the proportion of ‘guilty’ verdicts to the same degree. Hope et al.^6^ conducted two experiments comparing the two- versus three-verdict system and also found that the availability of the ‘not proven’ verdict reduced the proportion of ‘not guilty’ verdicts. Their results also showed that there was a small shift from guilty to not proven in ambiguous trials, that jurors were more confident in their verdicts in the three-verdict system and that 92% of jurors incorrectly believed that someone acquitted by a ‘not proven’ verdict would be treated differently from someone acquitted by a ‘not guilty’ verdict. Similarly, Curley et al.^7^ found that jurors were significantly less likely to give a ‘not guilty’ verdict in the three-verdict condition compared to the two-verdict condition, and that the ‘guilty’ verdict frequency was not significantly affected by the availability of the ‘not proven’ verdict. Their findings suggest that jurors interpreted the ‘not proven’ verdict to mean that ‘the evidence is not convincing enough to say guilty, but I don’t think they are innocent’.

Finally, Ormston et al.^2^ conducted a multifactorial experiment, looking not only at the verdict system (two vs. three) but also at jury size (15 vs. 12 jurors) and majority size (simple vs. unanimous). Jurors were presented with video vignettes (either rape or non-sexual assault) and gave individual verdicts before deliberating, group verdicts after deliberating and individual verdicts following deliberation. Jurors gave significantly fewer ‘guilty’ verdicts in the three-verdict system than in the two-verdict system (this finding only existed in non-sexual assault), which is in contrast to the findings of the studies outlined above. In acquittals, jurors were found to favour the ‘not proven’ verdict over the ‘not guilty’ verdict. Jurors in 12-person juries were found to participate more in jury deliberations. They were more likely to change their mind about a chosen verdict, and were significantly less likely (post deliberation) to favour a ‘guilty’ verdict than jurors in 15-person juries were. Jurors in the simple majority verdict condition were significantly more likely to give a ‘guilty’ verdict in comparison to jurors in the unanimous verdict condition, although this effect was only significant at the post-deliberation stage. Juries in the unanimous verdict condition also took longer to deliberate than juries in the simple majority condition did. Furthermore, jurors who had two verdicts available whilst participating in a 15-person jury and who could give a simple majority verdict were the most likely group to favour a ‘guilty’ verdict post deliberation, and jurors who were able to give one of three verdicts, based on a unanimous decision in a 12-person jury, were the group least likely to give a ‘guilty’ verdict post deliberation.

In summary, the studies outlined above have all highlighted that the legal structure of the jury system influences how jurors and juries make decisions.^2^ There is evidence that legal structures (e.g. verdict system) can interact with crime types to influence verdict choice,^6^ and that the unique aspects of Scottish trials may interact with each other to affect verdicts. However, a limitation of these studies for informing policy is that they do not highlight the legal value of increasing or decreasing the frequency of ‘guilty’/‘not guilty’ verdicts.

Legal stakeholders have the expertise and experience required to give an informed perspective on the impacts of changes in frequency of different verdicts. Their careers are built on understanding the checks and balances of the Scottish system that maximise the fairness of trials and minimise the possibility of miscarriages of justice. These elements will be tested through questions on reform and on methods of bias reduction and juror participation. Legal professionals may be involved in hundreds of jury trials over their careers, whereas most jurors encounter only one. Any changes to the system that might alter the frequency at which juries deliver particular verdicts are changes that should be interpreted alongside the attitudes of legal professionals in order to gain insight into whether these changes are positive or negative. They will be aware of changes to policy that may be seen to favour convictions or acquittals, and will have informed perspectives on whether either side of the Scottish adversarial system has an easier path during a trial than the other. Opinions from other groups (e.g. complainants, accused individuals, policymakers) should also be collected. However, due to the unique experience and knowledge that Scottish legal professionals have in the Scottish legal system, the current study will focus on their insights, as they are likely to give important context to the findings of previous experimental juror studies.

Policy recommendations regarding juror decision making should also not be based on experimental simulations alone.^8^ Simulations have poor ecological validity (experimental materials do not replicate real-life court processes), as mock jurors are presented with evidence through a screen rather than witnessing the evidence themselves in court.^8^ Legal professionals are therefore sceptical about how far findings from simulation studies apply to a real-world jury context.^8^ Consequently, Krauss and Lieberman^8^ suggest that researchers interested in juror decision making should utilise a number of methods, each with their own strengths and weaknesses, in an attempt to give more holistic conclusions regarding how jurors make decisions. For example, juror simulations lack ecological validity but do have strong internal control, whereas legal professionals have years of experience in relation to the legal system in Scotland and can aid with defining currently undefined verdicts, but their views will be biased by their own attitudes. If similar recommendations can be made through different methods, and findings cannot potentially be explained by the limitations of one method’s weaknesses alone, then policymakers will have a more nuanced and sophisticated understanding of what policy reforms should take place.^8^

### Current study

As researchers have focussed on experimental studies of juror/jury decision making, the views of legal professionals on the ‘not proven’ verdict, the 15-person jury system and the simple majority verdict are absent from the academic literature. The current paper aimed to address this by investigating how Scottish legal professionals view these three factors and by seeking guidance from these professionals on potential areas of reform. The research team sought to determine: (a) whether legal professionals view the availability of the ‘not proven’ verdict as a positive or negative influence in the jury system; (b) whether legal professionals have a preference for a particular jury size (15 vs. 12); (c) how legal professionals view the role of the majority size in how it influences jurors and juries; and (d) what suggestions they may have in regards to potential ways to reform the current Scottish jury system. Understanding legal professionals’ perceptions of each of the three factors discussed and their suggestions for potential reform is important for two main reasons. First, the information will provide legal value and insight to the findings of previous juror research.^2^ Second, potential policy changes to the Scottish jury system should take legal experience into account, and the current research will provide a bridge between legal professionals and policymakers. The current study has three main aims: (a) to inform debate surrounding each of the three main factors in this study; (b) to include the voice of legal stakeholders in academic research and potential policy change; and (c) to draw on the expertise of legal professionals who work on real cases to see if their views reflect the findings of controlled experimental studies.

## Method

### Design

An online survey was used to collect quantitative and qualitative (i.e. free-text) data on the attitudes of legal professionals in Scotland to the three-verdict system, 15-person jury and simple majority verdict system. The survey also asked about specific verdicts which are present in current verdict systems and alternatives that are not currently used. The study received ethical approval from the Open University’s Human Research Ethics Committee (reference HREC/3554).

### Participants

Participants were recruited through the Faculty of Advocates and Scottish Legal News and via Twitter. There were 78 participants (35 females, 43 males), aged between 24 and 76 years (*M* = 48.28 years; *SD* = 12.88 years) who submitted data; there are approximately 11,000 legal professionals in Scotland.^9^ Participants self-defined their ethnicity as white British/Scottish (*n* = 70), white other (*n* = 5) and Asian (*n* = 3); Scotland’s ethnic makeup is 96% white and 2.6% Asian.^10^

Twenty-one participants identified as solicitors, 16 as advocates and 22 as procurator fiscals. The remaining 15 participants identified as one of the following: trainee, judge, sheriff, solicitor advocate, non-practicing solicitor; lawyer or solicitor advocate judge. Four individuals did not identify their specific role within the legal system. Years of experience with the Scottish legal system ranged from 1 to 55 (*M* = 23.38 years; *SD* = 13.06 years).

### Materials

The questionnaire had five sections: general demographics/legal experience, the ‘not proven’ verdict, 15-person juries, simple/majority verdict systems and the general Scottish legal system. With the exception of the demographics section, all questions used a combination of forced-choice, open-ended (free text) and 10-point Likert-type scale responses. For those using Likert scales, most scales ranged from 1 to 10, with 1 rated as the least agreement or most negative response to the question and 10 as the most agreement/positive response. However, some questions were reverse scored.

There were 30 questions about the ‘not proven’ verdict. Fourteen items asked for a scale response to questions, such as ‘How do you perceive the “not proven” verdict in the Scottish legal system?’. Twelve items were forced-choice and four were open-ended questions. There were 27 questions about 15-person juries, of which 15 were scale items, 11 were forced-choice questions and one was an open-ended question. The one open-ended question in this section was ‘Please give your justification for the abolition or keeping of the 15-person jury system’. The simple versus majority verdict system was assessed with 36 questions, of which 18 were scale questions, 17 were forced-choice questions and one was an open-ended question. The forced-choice items in this section included ranking questions, such as ‘Please rank your preferred verdict system (with 1 being your most preferred system and 3 being your least preferred system)’ for ‘simple majority system’, “’unanimous system’ and ‘qualified majority system’. The final section of eight general questions consisted of four scale items, three forced-choice items and one open-ended item. The open-ended question asked for suggestions of things to be introduced or removed from the Scottish legal system.

### Procedure

Once participants consented to participate, they could respond to the questions and were allowed to skip any they chose not to answer. At any point in the survey, they could close the survey window, and their data would be retained for one week. If they did not return to the study, this was treated as a withdrawal, and their data were deleted. If they returned within one week, they were able to continue the survey where they left off. Upon reaching the end of the survey, participants were taken to a debriefing page.

## Results

The results are divided into four subcategories: (a) verdict system, (b) jury size, (c) majority size and (d) general jury system. The minimum number of participants who responded to a question was 55, and the maximum was 78.

### Verdict system

Participants were asked to rank their most preferred (rank 1), second most preferred (rank 2) and least preferred verdict system (rank 3) out of: (a) guilty, not guilty and not proven; (b) guilty and not guilty; and (c) not proven and proven. There was a significant difference in mean rank given to verdict types (χ^2^(2) = 57.47, *p* < 0.001). Post hoc analysis found a significant difference between mean ranks for each verdict type, with guilty and not guilty (mean rank = 2.41) ranked less favourably than either guilty, not guilty and not proven (mean rank = 1.96; *Z* = –4.69, *p* < 0.001) or proven and not proven (mean rank = 1.64; *Z* = –6.16, *p* < 0.001). Guilty, not guilty and not proven was ranked significantly less favourably than proven and not proven (*Z* = –4.00, *p* < .001). Slightly more respondents ranked proven and not proven as their most preferred option than the current three verdict system (30 vs. 28; 40.54% vs. 36.84%). Guilty and not guilty was ranked as the least preferred options by almost half of the respondents (*n* = 36; 48.65%).

Forty-five (60%) individuals suggested the legal system should keep the ‘not proven’ verdict, whereas 25 (33.33%) suggested abolishing the verdict; this finding was significant (binomial test result; *p* = 0.02). Qualitative reasons given for keeping the verdict included (a) the ‘not proven’ verdict better reflects the purpose of court (*n* = 17; 22.67%); (b) it gives jurors a way to express doubt without declaring a moral position (*n* = 9; 12%); (c) it reflects the non-binary nature of life decision making (*n* = 7; 9.33%); (d) elements of the legal system should not be changed in isolation without considering other elements of the system (*n* = 6; 8%); (e) it may encourage more ‘guilty’/‘not guilty’ verdicts (*n* = 3; 4%); and (f) juries prefer having it, it protects against biases or it provides a balance to crown advantages (*n* = 3; 4%). Reasons for abolishing the ‘not proven’ verdict include jury misunderstandings about the verdict (*n* = 11; 14.67%), that the function of the court is to make a binary choice (*n* = 6; 8%) and that it allows jurors a way out of making decisions about the fate of the accused (*n* = 8; 10.67%). Five (6.67%) participants gave responses to the question regarding the abolition of the ‘not proven’ verdict that left their preference unclear and/or provided a more nuanced answer. For example, one individual noted that ‘not proven’ was not justified in a three-verdict system, but would be suitable for a ‘proven or not proven’ two-verdict system.

In relation to perceived juror understandings of the ‘not proven’ verdict, 55 (75.34%) participants believed that jurors would perceive the verdict to mean ‘innocent in law but not community’, whereas 18 (24.66%) believed that jurors would perceive the verdict to mean ‘innocent in law and community’. Fifty-three (67.95%) participants perceived the ‘not proven’ verdict to reflect a situation where the case had not been proven beyond reasonable doubt, eight (10.26%) perceived it as being used by cautious juries instead of not guilty and six (7.69%) perceived it as a verdict used by juries who are just shy of reasonable doubt or are nervous about convicting. A further four (5.14%) participants believed ‘not proven’ reflected an acquittal or verdict of innocence, and seven (8.97%) participants provided answers that did not provide a clear definition. When asked to define a potential ‘proven’ verdict, 64 (82.05%) participants defined it as reflecting a situation where a crime had been proven beyond reasonable doubt, nine (11.54%) saw it as the same or close to the ‘guilty’ verdict and three (3.85%) saw it as the same as or close to the ‘not guilty’ verdict. Two (2.56%) participants did not give a clear definition. Several further questions were also asked to test participants’ perceptions of the Scottish jury system (see Table 1).

**Table 1.** Mean ratings (on a scale of 1–10) and standard deviations for perceptions of the ‘not proven’ verdict.

Questions	Mean	Standard deviation
Perception of three-verdict system	6.18	6.12
Perception of ‘not proven’ verdict	6.11	3.54
Perceived juror understanding of ‘not proven’ verdict	4.85	3.08
Perceived accuracy of juror usage of ‘not proven’ verdict	5.77	3.00
Position on abolishment of ‘not proven’ verdict (reverse scored)	6.06	3.80
Perception by general public on accused receiving ‘not proven’ verdict (reverse scored)	5.41	2.70

### Jury size system

**Figure 1.**
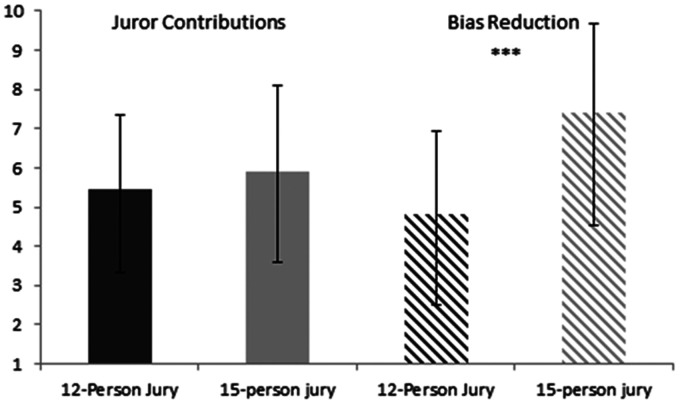
Mean ratings for the perceived effect of 15-person and 12-person juries on increasing juror contributions and reducing biases. Error bars show standard deviations, and *** indicates significant differences.

When asked to indicate their preferred jury size system (15 vs. 12), 63 (85.14%) participants preferred the 15-person jury size, whereas 11 (14.86%) preferred the 12-person jury size; this finding was statistically significant (binomial test result; *p* < 0.001). When asked about keeping or abolishing the 15-person jury system, 52 (70.27%) favoured keeping the 15-person jury, and gave reasons such as it allows a more representative jury to be collected (*n* = 20; 27.03%) and that it leads to less bias and more flexibility for drop-outs, and that there is no evidence that smaller jury sizes are better (*n* = 7; 9.46%). Many participants qualified their preference to keep the 15-person jury by indicating conditions to their preference. Sixteen (21.62%) participants indicated that the number of jurors was less important than the majority system used, and nine (12.16%) suggested it was not possible to change juror numbers without also changing other elements of the legal system. Six (8.11%) participants favoured abolishing the 15-person jury system, suggesting that a larger group size increases the chance for a group mentality, or that sufficient jurors are sometimes difficult to find. Significantly more participants preferred to keep the 15-juror system than abolish or change it (binomial test result; *p* < 0.001). Six (8.11%) participants suggested that juries should be removed and replaced by a panel of judges, six (8.11%) suggested that the jury size made no difference to them and four (5.41%) gave answers which did not clearly state a preference or indicated neutrality on the issue. Several further questions asked about participants’ perceptions of the current 15-person jury system (see Table 2 and Figure 1).

**Table 2.** Mean ratings (on a scale of 1 to 10) and standard deviations for perceptions of the 15-person jury.

Questions	Mean	Standard deviation
In favour of keeping 15-person jury	7.93	2.53
How positively do you see the current 15-person system?	7.38	2.60
Current position on the abolition of 15-person jury system (reserve scored)	7.36	3.04

### Majority size

Participants were asked to rank their most preferred (rank 1), second most preferred (rank 2) and least preferred majority size out of: (a) simple majority, (b) qualified majority and (c) unanimous. There was a significant difference in mean rank given to majority sizes (χ^2^(2) = 77.06, *p* < 0.001). Post hoc analysis revealed a significant difference between mean ranks for each majority size, with the unanimous verdict size (mean rank = 2.75) being ranked less favourably than either simple majority (mean rank = 1.81; *Z* = –5.64, *p* < 0.001), or qualified majority (mean rank = 1.44; *Z* = –6.36, *p* < 0.001). The simple majority size was ranked significantly less favourably than the qualified majority size (*Z* = –3.87, *p* < 0.001). The much greater part of the sample preferred either qualified majority (*n* = 27; 49.09%) or simple majority (*n* = 23; 41.82%), while most participants ranked the unanimous majority size as their least preferred option (*n* = 38; 69.09%).

**Figure 2.**
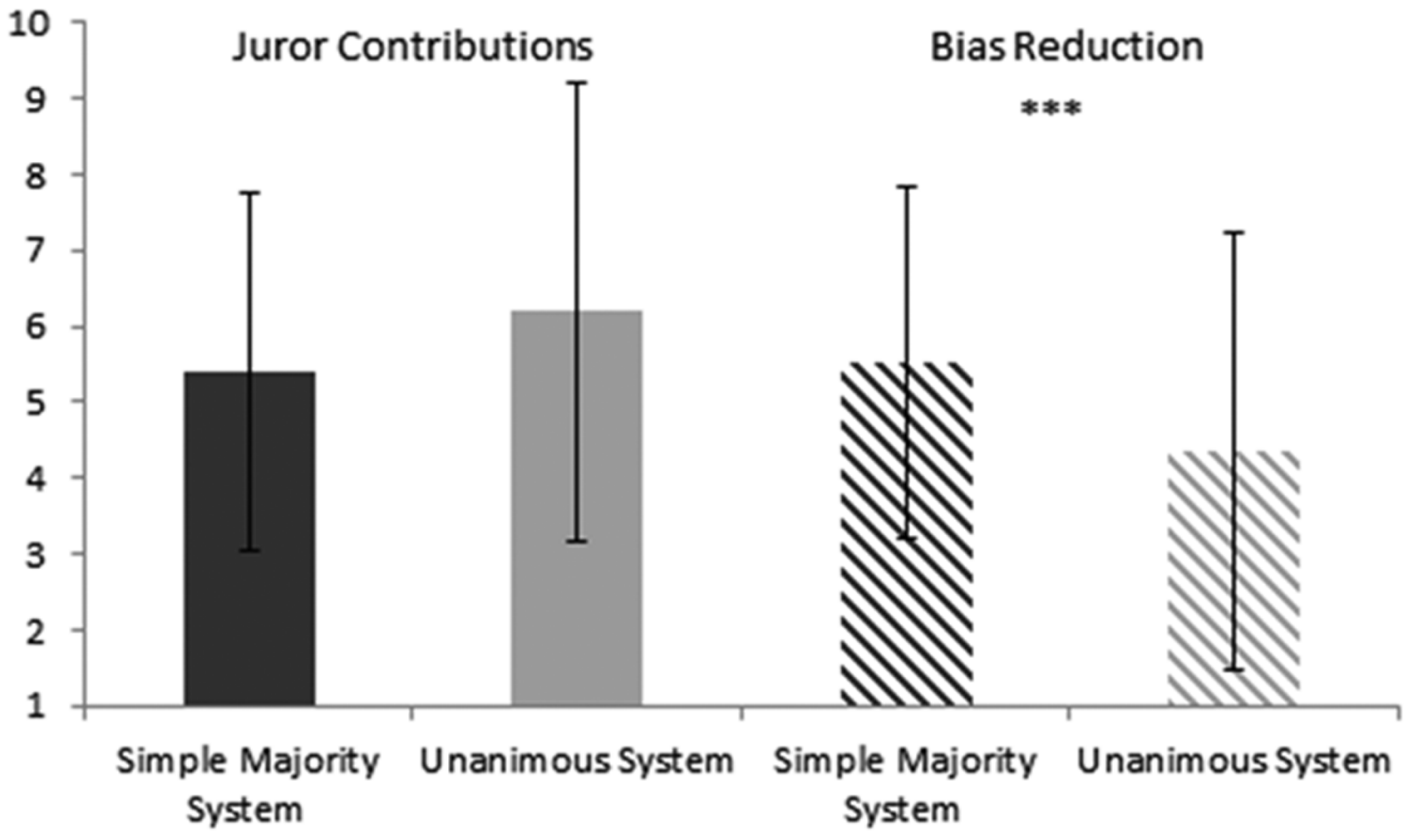
Mean ratings of the effect of simple majority and unanimous majority systems on increasing juror contributions and reducing biases. Error bars show standard deviations, and *** indicates significant difference.

Forty-one (60.29%) participants suggested that the simple majority system should be kept, with the given reasons being: (a) the system is currently working fine (*n* = 17; 25%); (b) it promotes discussion and/or speed and avoids a hung jury (*n* = 8; 11.77%); (c) it provides power to individual jurors (*n* = 7; 10.29%); and (d) unanimity can be difficult to achieve, it gives a needed advantage to the prosecution or abolishing the simple majority system would lead to more acquittals (*n* = 5; 7.35%). Four (5.88%) participants stated that they would keep the simple verdict system if pressed but would prefer the removal of juries altogether. However, 21 (30.88%) participants suggested that the simple majority verdict should be abolished, 14 (20.59%) said that it was too small a majority for such a big decision and seven (10.29%) stated that the qualified majority system is the fairest. Significantly more participants preferred to keep the simple majority system (binomial test result *p* = 0.004). There were three (4.41%) answers that suggested the majority size could not be changed without cascading changes to other protections in the system, or that it depended on who was being asked. Three (4.41%) participants gave answers that did not clearly indicate a preference. Table 3 and Figure 2 demonstrate answers to further questions asked about the impacts of different majority sizes.

**Table 3.** Mean ratings (on a scale of 1 to 10) and standard deviations for perceptions of the simple majority.

Questions	Mean	Standard deviation
In favour of keeping simple majority verdict system	6.86	3.02
Current position on abolition of simple majority verdict (reverse scored)	6.71	3.15

### General jury system

Participants were asked if they were designing the legal system, which options they would choose for each of the three factors (verdict system, jury size and majority size). In terms of verdict system, 16 (20.51%) participants said that they would choose a guilty and not guilty system, 22 (28.21%) would choose a guilty, not guilty and not proven system, and 40 (51.28%) would choose a proven and not proven system; this finding was significant (χ^2^(2) = 12.00, *p* = 0.002). In terms of jury size, 13 (17.33%) participants said that they would use a 12-person jury, whereas 62 (82.67%) would choose a 15-person jury (binomial test result; *p* < 0.001). In terms of majority size, 42 (55.26%) participants said that they would choose a simple majority verdict system, 30 (39.47%) would choose a qualified verdict system (10 out of 12, or 12 out of 15) and four (5.26%) would choose a unanimous verdict system (χ^2^(2) = 29.79, *p* < 0.001). Table 4 gives details on participants’ preferred combinations of verdict.

**Table 4.** Preferred combinations of three aspects of a legal system: jury size, majority type and verdict system (number of votes and percentage of total votes).

Combinations	Frequency	%
15 jurors, simple majority, proven/not proven	16	21.33
*Status Quo: 15 jurors, simple majority, guilty/not guilty/not proven*	13	17.33
15 jurors, qualified majority, proven/not proven	11	14.67
15 jurors, simple majority, guilty/not guilty	10	13.33
15 jurors, qualified majority, guilty/not guilty/not proven	7	9.33
12 jurors, qualified majority, proven/not proven	6	8.00
15 jurors, qualified majority, guilty/not guilty	3	4.00
12 jurors, unanimous verdict, proven/not proven	2	2.67
12 jurors, qualified majority, guilty/not guilty/not proven	1	1.33
12 jurors, simple majority, guilty/not guilty	1	1.33
*Anglo-American System: 12 jurors, unanimous, guilty/not guilty*	0	0
All other combinations	0	0

Several further questions were asked about participants’ general perceptions of the Scottish jury system. Table 5 summarises the responses. Frequencies and percentages of participants’ beliefs about which side particular elements of the Scottish legal system favour were also measured (see Table 6).

**Table 5.** Mean ratings (on a scale of 1 to 10) and standard deviations for perceptions of the Scottish jury system.

Questions	Mean	Standard Deviation
Current legal system’s ability to provide justice to victims	6.53	2.47
Current legal system’s ability to provide justice to accused	6.76	2.34
How much confidence do you have in the current legal system?	6.88	2.39
How do you feel about current Scottish legal system being reformed to become more like Anglo-American system?	2.92	2.07

**Table 6:** Frequencies and percentages of participants’ beliefs about which side particular elements of the Scottish legal system favour.

	Favours defence		Favours neither side		Favours prosecution	
	*n*	%	*n*	%	*n*	%
*‘Not proven’ verdict*						
Inclusion*	42	53.85	36	46.15	0	0
Removal*	7	8.97	40	51.28	31	38.74
*Jury size*						
15-person*	8	10.53	63	82.9	5	6.85
12-person*	18	24	52	69.33	5	6.67
*Majority size*						
Simple majority*	57	74.03	20	25.97	0	0
Qualified majority*	45	58.44	31	40.26	1	1.3
Unanimous*	3	3.9	45	58.44	29	37.66

*Significant deviation from equal distribution according to a one-sample chi-square test.

## Discussion

Scottish legal professionals have a unique perspective on the Scottish legal system, and an unmatched exposure to it throughout their careers. Most mock jurors may never experience a real courtroom, whereas legal professionals may have decades of relevant experience. The current study represents the only existing academic investigation into legal professionals’ views on the Scottish legal system to date. This discussion follows the following structure: (a) verdict system, (b) jury size, (c) majority size and (d) general policy implications and conclusions.

### Verdict system

The main finding in relation to the preferred verdict system was that participants ranked their most preferred system to be ‘proven and not proven’ and their least preferred system to be ‘guilty and not guilty’. The majority of participants also suggested that the ‘not proven’ verdict should be kept. In addition, there was a tendency for participants to view the three-verdict system positively and to be against the abolition of the ‘not proven’ verdict. Further, the availability of the ‘not proven’ verdict was seen to favour the defence, and its removal was seen to favour the prosecution. This is consistent with Ormston et al.’s^2^ findings and indicates that legal professionals agree with the findings of that study, as they suggest that the removal of ‘not proven’ may increase the frequency of ‘guilty’ verdicts. Three other studies using mock jurors found that the ‘not proven’ verdict did not favour the prosecution, however,^5–7^ so the legal professionals’ views were inconsistent with those findings.

Previous research has highlighted that the availability of the ‘not proven’ verdict can significantly decrease the proportion of ‘not guilty’^5,7^ and ‘guilty’ verdicts^2^ given. Juror simulation research cannot assess whether this reduction is positive or a negative when considering the aims of the legal system (i.e. maximising fairness and minimising miscarriages of justice). Insight from legal professionals, who are well informed about elements of the system that may support the prosecution or the defence, is therefore vital when evaluating the legal value of changes in verdict frequencies. Qualitative data from the current study suggest that the availability of the ‘not proven’ verdict (and consequent reduction in ‘guilty’ and ‘not guilty’ verdicts) may have a positive effect, as it allows jurors to express doubt without declaring a moral position, allowing the court to reflect its own purposes more accurately. For instance, the courtroom does not deal in principles of absolute truths. Thus, terms such as ‘guilty’ and ‘not guilty’ may mislead legal novices^10^ such as jurors or members of the general public reading about trials in newspapers. Terms such as ‘proven’ may therefore hint to jurors the true purposes of court (i.e. to highlight if a crime has been proven with the evidence to hand^10^).

When asked what they would do if they could redesign the Scottish legal system from scratch, most participants indicated that they would use a binary-verdict system of ‘proven’ and ‘not proven’. This suggestion may link (once again) to ideas surrounding proof and truth, and may direct jurors to their true role in a more nuanced way. The role of a jury is not to decide on whether an accused did factually commit the crime. Rather, their job is to use the evidence to establish whether the prosecution has proved their case beyond reasonable doubt.^10^ When asked, the vast majority of our sample of legal professionals defined the ‘proven’ verdict to mean that a crime ‘had been proven beyond reasonable doubt’. This definition had greater consensus than any of the existing verdicts in the Scottish legal system.

The current study also highlighted that participants tended to perceive juror understandings of the ‘not proven’ verdict to be poorer than their ability to use the verdict accurately. This is consistent with the findings of previous juror experiments, as Hope et al.^6^ found that 55% of their sample in their two-verdict condition had never heard of the ‘not proven’ verdict and so were unlikely to have a concrete idea of its meaning and consequences; this does not mean that they would not have used it as an aquittal verdict if it were availiable to them and they were directed by a judge, however. Further, Ormston et al.^2^ found that jurors struggled to differentiate the ‘not proven’ verdict from the ‘not guilty’ verdict, being unsure about the consequences for someone acquitted by a ‘not proven’ verdict. It has been suggested that jurors give ‘not guilty’ verdicts when they believe that the accused is innocent, whereas they give ‘not proven’ verdicts when they believe the prosecution failed their case.^2,7,12^ This suggestion may link with the findings of the current study as the majority of participants believed that jurors would view accused individuals who had recieved a not proven verdict as being 'innocent in law but not community'. 

Jurors’ lack of understanding of the ‘not proven’ verdict may not justify its removal, but rather it may provide a reason to define the verdict formally. The vast majority of participants in the current study suggested that the verdict be defined to mean ‘not proven beyond reasonable doubt’, which would provide a clear counterpoint to the definitions of the (currently unused) ‘proven’ verdict given by our participants. Without input from legal professionals, it would be difficult to define the ‘not proven’ verdict clearly in a way that accurately represents its legal purpose.

### Jury size

The main finding in relation to jury size was that participants were satisfied with the current 15-person jury system, preferring it to the 12-person jury system and indicating that they would choose it if they were redesigning the legal system. Participants believed that the 15-person jury system was more effective than a 12-person jury at attenuating biases. The current study did not find a significant difference in participants’ perceptions of individual juror contributions in the 15-person and 12-person juries. This latter point deviates from research conducted by Ormston et al.,^2^ who found that juror contributions were decreased in 15-person juries compared to 12-person juries. Future research is clearly needed here, as there are limitations to both the Ormston et al.^2^ paper and the current study. First, the maximum limit of deliberation in Ormston et al.’s^2^ study was 90 minutes, and such a time limit may have limited the abilities of certain jurors to participate in discussions. Second, legal professionals do not have access to jurors and may overestimate the part that each juror plays in the deliberation process.

A small minority of participants in the current study suggested that juries be abolished and replaced by a panel of judges. Previous research would suggest that this recommendation would not help improve the system, as legal professionals have been consistently shown to be susceptible to similar biases and errors as jurors.^13,14^

Previous research has highlighted that jurors are more likely to favour the ‘guilty’ verdict (and thus the prosecution) in a 15-person jury when compared to a 12-person jury.^2^ Such a finding when interpreted alongside some of the reasons given by participants in the current study for keeping the 15-person jury may highlight that the usage of 15-person juries may be increasing the likelyhood of a ‘true conviction' being given. For instance, participants stated that 15-person juries (a) allow a more representative jury to be created, and (b) lead to an attenuation of bias in individual jurors. Further, through the jury being larger, a wider representation of beliefs and biases is likely, which may then cause an attenuation in the influence that bias plays in verdict making.^15^ Therefore, an increase in convictions when comparing 15-person juries with 12-person juries may be highlighting that the former decision is more objective in assessing the evidence, rather than being biased towards the prosecution; this may be useful to policymakers when considering potential reforms.

### Majority size

Most of the participants in the current study ranked the qualified majority verdict (either a 10/12 version or a 12/15 version) as their most preferred majority size, although simple majority was a close second. The unanimous verdict system was voted as the least preferred verdict system by the majority of participants. However, the majority of participants in our study suggested that they would choose a simple majority if they could design the legal system from scratch. This may reflect a perception amongst legal professionals that the qualified majority system is preferred as part of the current system, but that a simple majority would be optimal alongside changes to other parts of the Scottish legal system.

The majority of participants perceived both the unanimous and qualified verdict systems to favour the defence and that the simple majority verdict system favoured neither side. Previous research with jurors has also highlighted that jurors working with a simple majority verdict are more likely to favour a ‘guilty’ verdict than jurors working with a unanimous verdict.^2^ The results also highlighted that participants were currently against abolishing the simple majority verdict for a unanimous verdict. Reasons given for keeping the simple majority verdict included promoting discussion and speed of decision making and avoiding a hung jury, and there being no need to change the current system. Participants indicated that the simple majority verdict was better at reducing juror biases but was no better at increasing juror contributions than a unanimous verdict. Ormston et al.,^2^ however, found in their study that requiring unanimous verdicts led to significantly more participation by jurors and longer deliberations. If a requirement for a larger proportion of the jury to agree leads to more juror participation (as shown in Ormston et al.’s^2^ study), a potential compromise to ensure efficient deliberations, a decreased chance of hung juries and satisfied Scottish legal professionals may be for the jury system in Scotland to adopt a qualified majority verdict system.

### General jury system preferences

When asked what elements of the Scottish legal system they would choose if they could redesign it, the most popular combination selected by legal professionals in the current study was a ‘proven or not proven’ verdict system, a 15-person jury and a simple majority verdict size. The second most popular option was the current system, which may highlight a status quo bias. The majority of the sample was reflected in the top 3 most popular combinations, with the third most popular being ‘proven or not proven’, 15-person jury and a qualified majority size. Interestingly, the Anglo-American system of ‘guilty or not guilty’, a 12-person jury and a unanimous majority size was the least popular combination, with no supporters. In general, the participants in the current study showed high confidence in the Scottish legal system and felt negatively about reforming the system to be more like the Anglo-American system.

## Conclusion

In conclusion, from involving stakeholders in research on the ‘not proven’ verdict, the 15-person jury system and the simple majority system, a number of recommendations can be made. First, legal professionals show a preference for reforming the current three-verdict system to a binary-verdict system where the options are ‘proven’ and ‘not proven’; this finding is one that would be unlikely to come from experimental jury research. Second, legal professionals strongly prefer that the 15-person jury system is kept, and see it as a method of reducing bias when compared to a 12-person jury. Third, legal professionals prefer a qualified majority verdict system (12/15) and are strongly against a unanimous verdict system. All these findings are novel to the literature. Furthermore, the current study has found novel findings (i.e. that legal professionals would prefer a verdict system of ‘proven’ and ‘not proven’), and can make some similar conclusions to Ormston et al.,^2^ despite using different legal actors (legal professionals rather than jurors) and varying methods (survey vs. jury simulations).^7^ By triangulating the data from different studies, using a variety of methods and different participants in the legal process, conclusions can have greater utility to policymakers.^7^ For instance, taken together, the current study and the study by Ormston et al.^2^ indicate that (a) a binary-verdict system of ‘proven’ and ‘not proven’ would be preferred by legal professionals, who feel that it would provide greater access to justice, but (b) a binary-verdict system may increase convictions overall relative to a three-verdict system. Similarly, legal professionals’ preferred option of a 15-person jury system may increase convictions in comparison to a 12-person jury system, whereas the preferred option of a qualified majority verdict system may increase the number of acquittals in comparison to a simple majority verdict system. However, Ormston et al.’s^2^ research did not consider a ‘proven’ versus ‘not proven’ system with a qualified majority, thus not including experimental conditions that reflect the preferred model of legal professionals. In order to inform policy most usefully, a further juror simulation study is needed which includes these conditions in its experimental design (indeed, such a study would usefully be informed by consulting with legal professionals at the design stage). Crucially, a clear definition of what the ‘not proven’ verdict means is necessary for juries to perform their task.
